# Mortality due to sepsis in Brazil in a real scenario: the Brazilian ICUs project

**DOI:** 10.5935/0103-507X.20190008

**Published:** 2019

**Authors:** Suzana Margareth Lobo, Ederlon Rezende, Ciro Leite Mendes, Mirella Cristinne de Oliveira

**Affiliations:** 1 Unidade de Terapia Intensiva, Hospital de Base, Faculdade de Medicina de São José do Rio Preto - São José do Rio Preto (SP), Brasil.; 2 Unidade de Terapia Intensiva, Hospital do Servidor Público Estadual, Instituto de Assistência Médica ao Servidor Público Estadual - São Paulo (SP), Brasil.; 3 Unidade de Terapia Intensiva, Hospital Universitário, Universidade Federal da Paraíba - João Pessoa (PB), Brasil.; 4 Unidade de Terapia Intensiva, Hospital do Trabalhador - Curitiba (PR) Brasil.

Worldwide, the number of sepsis patients per year is estimated at 15 to 17 million, contributing to more than 5 million deaths annually.^(^^1-3^^)^ In Brazil, recent publications have indicated an increase in the number of cases of this syndrome in late years.^(^^4^^)^ Many factors have contributed to this trend, such as population growth and rising in life expectancy, which rose from 65.3 years in 1990 to 71.5 years in 2013, increasing the susceptible population to include elderly people, people with chronic diseases, and immunosuppressed people.^(^^5^^)^ In addition, initiatives such as the Sepsis Survival Campaign (CSS) and the Global Sepsis Alliance (GSA) are some means used in better identifying septic patients and increasing disease reporting.

The Brazilian ICUs project, created by Epimed Solutions^®^, together with the *Associação de Medicina Intensiva Brasileira* (AMIB) (http://www.utisbrasileiras.com.br/project/), is based on the National Registry of Intensive Therapy, with the objective of characterizing the epidemiological profile of Brazilian intensive care units (ICUs) and sharing epidemiological information that may be useful in guiding public health policies and developing research and treatment strategies to improve the outcomes of critically ill patients in Brazil.^(^^2,4^^)^ The participation of ICUs in the Epimed Database is voluntary and governed by a commercial agreement with Epimed Solutions^®^, an information technology company responsible for the development, updating, security, and backup of all processes.^(^^4^^)^ The participants in the Brazilian ICUs project have access to a free and simplified version of the system.

The purpose of this review was to disclose the temporal trends of sepsis prevalence and mortality. We evaluated data from a large national registry, with participation of approximately 30% of the adult ICU beds in the country, with data from 190,999 hospitalized patients between 2010 and 2016 in 638 ICUs from 349 public and private hospitals that were part of the Brazilian ICUs project. All of the Brazilian regions are represented, namely, 58.2% in the Southeast, 14.6% in the Northeast, 13.3% in the Midwest, 9.6% in the South, and 4.5% in the North.

**Figure 1:**
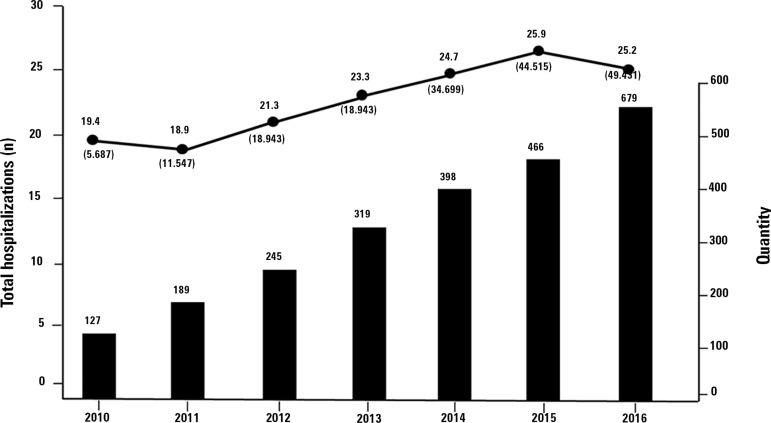
Temporal evolution of hospitalizations for sepsis (●) from 2010 to 2016 (n = 190,999) and number of intensive care unit participants (black bars).

**Figure 2:**
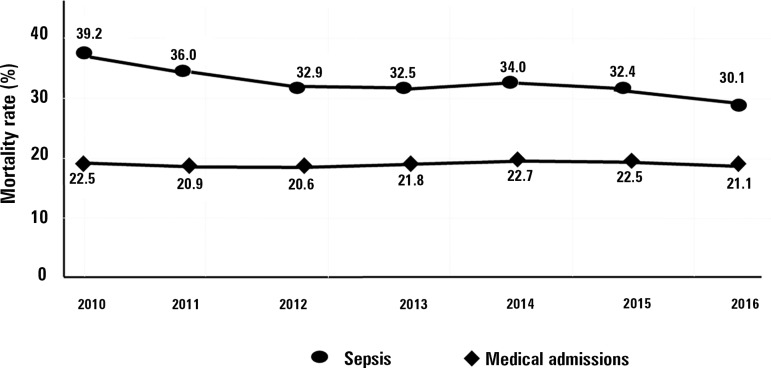
Time evolution of the hospital mortality rate of sepsis and other medical hospitalizations from 2010 to 2016 (absolute risk reduction of 9.1% (95%CI 7.7 - 10.4%, p < 0.001).

**Figure 3:**
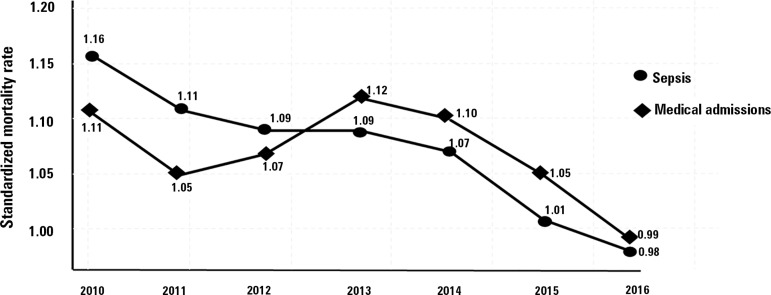
Temporal evolution of the standardized hospital mortality rate for sepsis and other medical hospitalizations from 2010 to 2016.

These data demonstrate a progressive increase in the number of cases of sepsis in Brazilian ICUs, from 19.4% of total hospitalizations in 2010 to 25.2% in 2016 (Figure 1), in addition to a stable and constant decrease in mortality. Mortality rates fell from 39% in 2010 to 30% in 2016 (absolute risk reduction - ARR: 9.1%, 95%CI 7.7 -10.4%, p < 0.001) (Figure 2) in patients with sepsis, while they remained unchanged for other medical hospitalizations. Standardized Mortality Rates (SMR), i.e., corrected for disease severity by the Simplified Acute Physiologic Score III, declined in the same period in patients with sepsis (Figure 3), and the overall rate in this 6-year period was 0.98 in private hospitals and 1.34 in public hospitals.

Brazilian studies performed between 2001 and 2003 showed mortality rates ranging from 34.4% to 34.7% in patients with severe sepsis and from 52.2% to 65.3% in patients with septic shock.^(^^6,7^^)^ SPREAD was a multicenter study conducted by the *Instituto Latino Americano da Sepse* (ILAS) that evaluated the prevalence and lethality of severe sepsis and septic shock in 2015.^(^^8^^)^ The distribution of Brazilian ICUs was obtained by consulting the AMIB census of ICUs, in which 1,813 ICUs (20,731 beds for adult patients) were registered in all Brazilian regions. Of these, 229 institutions included 794 patients, with a prevalence of 29.6% and an overall lethality of 55%. Mortality in the Southeast Region was 51.2%, i.e., lower than that in the other regions (Central West, 70%; Northeast, 58.3%; South, 57.8%; and North, 57.4%), and the mortality in the hospitals belonging to the public health system was no different from that which occurred in ICUs of the private system. While most ICUs were public (58%) in the SPREAD study, in the current analysis, the majority belonged to the private system (73%).

Implementing quality programs with education and care bundles can reduce mortality and is cost-effective.^(^^8,9^^)^ In an analysis of 21,103 cases (2005 - 2014) in the ILAS database, mortality reduction was significant in private hospitals (47.6% to 27.2%); however, this was not seen in public hospitals (61.3% to 54.5%). This difference is not likely to be related to the type of hospital financing (public *versus* private), but rather to their levels of organization, as evidenced by the quality of the processes and the greater adherence to the 6-hour package in private hospitals, which increased from 13.5% to 58.2%. This result is in contrast to what occurred in public hospitals, in which there was an increase in adherence rate of only 7.4% to 15.7%.^(^^9^^)^

Any project associated with quality improvement in an ICU requires actions focused on three key points, according to Avedis Donabedian: structure, processes, and results.^(^^10^^)^ The use of a database, such as the Epimed Database, makes it possible to transform data into information, which is fundamental for the good management and organization of a unit. However, daily inclusion of data is time-consuming, as is the interpretation of reports and the implementation of actions based on them. The ICUs that collected the data used in this manuscript possibly have reasonable levels of organization, which is an essential requirement for the maintenance of information used to feed a database of this size. Thus, it seems more appropriate to differentiate the ICUs by their level of organization than by their sources of funding, i.e., public or private.

While progressive declines in mortality rates are encouraging, differences among hospitals are worrying, and public health policy efforts should focus on management improvements. Stimulating better organization, particularly in public ICUs of a health system that suffers from a lack of resources and poor distribution of vacancies, should be part of public health policies. Our Brazilian ICUs program plays an important role, providing essential data to achieve a better understanding of the sepsis scenario in Brazil.
